# The Inguinal Herniation of the Ovary in the Newborn: Ultrasound and Color Doppler Ultrasound Findings

**DOI:** 10.1155/2014/281280

**Published:** 2014-03-26

**Authors:** Omer Kaya, Kaan Esen, Bozkurt Gulek, Cengiz Yilmaz, Gokhan Soker, Onder Onem

**Affiliations:** ^1^Department of Radiology, Numune Teaching and Research Hospital, 01240 Adana, Turkey; ^2^Department of Radiology, Faculty of Medicine, Namik Kemal University, 59100 Tekirdag, Turkey; ^3^Department of Pediatric Surgery, Numune Teaching and Research Hospital, 01240 Adana, Turkey

## Abstract

Inguinal hernias in the newborn age group are seldom encountered. In the affected female patient, the ovaries, fallopian tubes, and the intestines may settle in the hernia sac. The early diagnosis of torsion in cases in which the ovary is herniated into the inguinal canal is of utmost importance in order to give surgery the chance of reduction and correction. In this paper, a case of an ovarian herniation into the inguinal canal without the presence of torsion is being presented, and the place of US and CDUS in the differential diagnosis of the situation is being discussed.

## 1. Introduction

Inguinal hernias of the newborn era are encountered with a frequency of 1-2%, and the female/male ratio ranges between 1/4 and 1/10 [1]. In 15–20% of the female patients with inguinal hernias, the herniation sac may contain the ovaries and/or the fallopian tubes [2]. Despite the possibility of a spontaneous regression in some cases [3], the presence of ovaries and/or intestinal structures in the inguinal sac decreases the chance of regression, while also increasing the chance of incarceration [4–6]. Ovarian ischemia may arise in case the pedincule of the herniated ovary rotates around itself. Because of this reason, an early diagnosis of the situation is crucial in order to salvage the ovary before an irreversible damage happens. US and CDUS are the methods of priority in this early diagnosis. In this paper, a case of a herniated ovary in a female newborn which was diagnosed with US and CDUS before torsion took place is being presented.

## 2. Case Report

**Figure 1:**
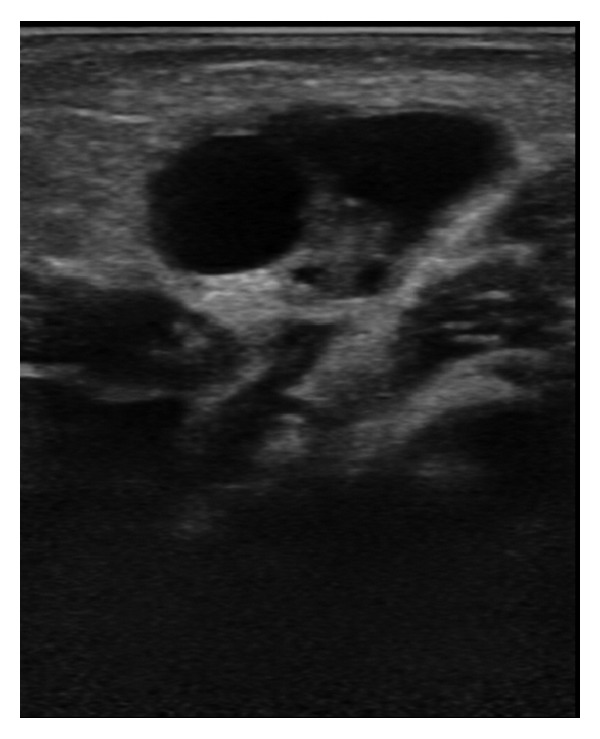
This B-mode US image shows the right ovary containing follicular cysts, in the right inguinal region.

**Figure 2:**
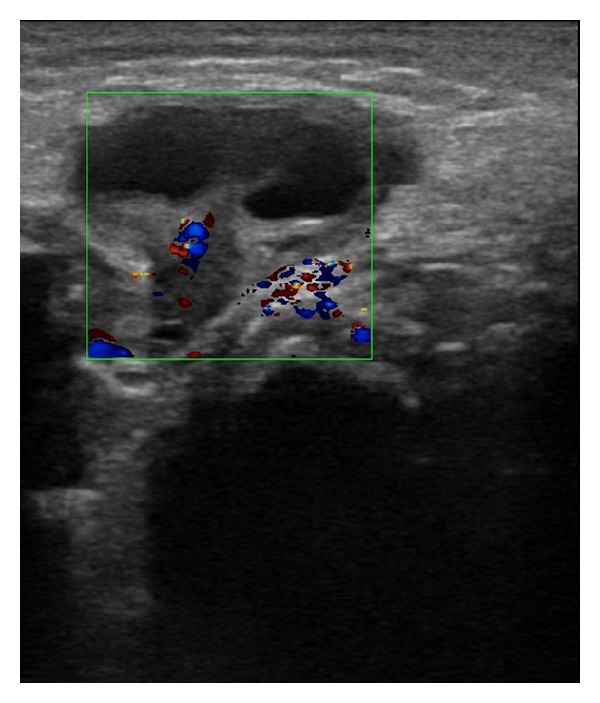
Arterial and venous flows in the right ovary at CDUS (arterial flow coded in red and venous flow in blue colors).

**Figure 3:**
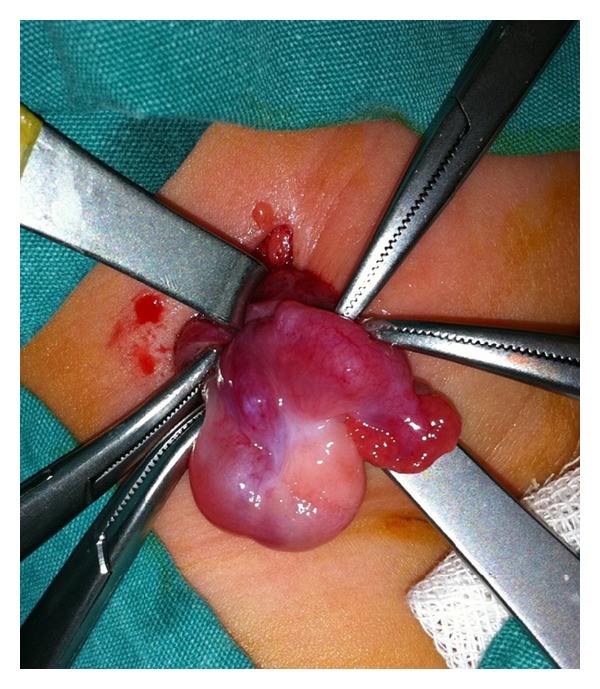
This intraoperative photo well demonstrates the right ovary and the fallopian tube in the hernial sac.

A female newborn of 62 days of age, who was born at the 39th gestational week with a birth weight of 3000 g, was brought to the pediatric outpatient clinic following the discovery of a lump in her right groin by her mother. The baby was anxious and she was in a steady state of intense crying. At physical examination, a tender mass was palpated in the right inguinal region, just above the labium majus. At B-mode US, a 7 mm wide fascial defect and a herniation of tissue material through this defect were detected. The herniated material included cystic structures of which the largest was about 10 mm in diameter, and suggestive of follicular cysts. Thus, this was diagnosed as the right ovary herniated through the inguinal canal (Figure 1). At CDUS, vascular signals were obtained from the ovarian tissue, thus indicating vitality, and leading to a nonconsideration of ovarian torsion (Figure 2). The baby was operated by the pediatric surgeon, and at the operation, an ovary herniated into the right inguinal canal but still not torsioned was visualized (Figure 3). A surgical procedure of ovarian reduction and high inguinal ligation was performed, and the hernial sac was fixed.

## 3. Discussion

The development of the inguinal canal is associated with two important anatomical structures, the gubernaculum testis and the processus vaginalis [7, 8]. The gubernaculum testis is the structure that enlarges in response to the increase in the hyaluronic acid content in the inguinal canal and thus widens the inguinal canal and the scrotum so that the testis can access a passage to pass through these organs. On the other hand, the processus vaginalis is the name of the invaginations which pass through the gubernaculum testis and extend into the scrotum after exiting the inner circle. In the female, the counterpart structure of the processus vaginalis which extends into the inguinal canal is known as the Nuck diverticulum [9]. The persistence of this peritoneal opening is defined as the Nuck cyst [10]. This peritoneal sac usually gets obliterated by the 8th gestational month [7]. Anomalies in the nonobliterated canal may lead to the development of inguinal hernias [10]. In prematurity situations, the delivery is accomplished before the closure of this canal, thus increasing the risk of the development of an inguinal hernia [9]. In addition, it has been reported that the risks of herniation and torsion are increased in cases in which the fallopian tubes are rather long and thus the ovaries more mobile [11]. Some lung problems, together with forceful stranding in long-standing constipation and vigorous crying, have all been held responsible for the increased risk of herniation due to increased intra-abdominal pressure [9]. It has been reported in a series of 211 inguinal herniation cases [4], all of whom were female newborns, that 27% of the cases were premature.

Inguinal hernias may contain the intestines, omentum, testes, ovaries, and fallopian tubes [12]. These structures may incarcerate. It has been reported that the most important complication of inguinal hernias in the pediatric age group is incarceration, which was found in a study to have a frequency of 31% [13]. The ovaries come first among the structures that incarcerate in the inguinal hernia sac. In a series of 1000 inguinal hernia cases, ovarian incarceration was reported to be present in 43% of the cases [14]. In another study done by Boley et al., it was reported that all of the 15 cases in the series had inguinal hernia sacs that contained nonreducible ovaries and that none of the sacs contained intestinal ingredients [5].

The importance of high-resolution US is outstanding in the diagnosis of inguinal herniations, just as it is in other superficial site examinations. The 3–5 MHz convex transducers used in routine pelvic US examinations are not satisfactory for the examination of the inguinal herniation sac, and it is recommended that a 5-MHz or higher frequency linear transducer be utilized for this purpose [1]. In a herniated ovary case, the increase in the ovarian dimensions, together with the heterogeneity of the ovarian echo structure, and the presence of peripherally located multiple cysts are all gray-scale B-mode US findings suggestive of ovarian torsion [13]. But the presence of these findings alone is not sufficient for a satisfactory evaluation of the ovary for the possibility of torsion and incarceration. It is the tool of CDUS which permits the examiner to evaluate the vascular structures at the ovarian pedincule and determine if the herniated and torsioned ovary tissue has suffered ischemia or not [13].
